# Multivariate Training Programs during Physical Education Classes in School Context: Theoretical Considerations and Future Perspectives

**DOI:** 10.3390/sports10060089

**Published:** 2022-06-03

**Authors:** Avelino Silva, Ricardo Ferraz, Pedro Forte, José E. Teixeira, Luís Branquinho, Daniel A. Marinho

**Affiliations:** 1 Department of Sport Sciences, University of Beira Interior, 6201-001 Covilhã, Portugal; avelinodasilva@gmail.com (A.S.); marinho.d@gmail.com (D.A.M.); 2 Research Center in Sports Sciences, Health Sciences and Human Development, 6201-001 Covilhã, Portugal; pedromiguel.forte@iscedouro.pt (P.F.); jose.eduardo@ipb.pt (J.E.T.); luis_branquinho@outlook.pt (L.B.); 3 Sport Department, Higher Institute of Educational Sciences of the Douro, 4560-408 Penafiel, Portugal; 4 CI-ISCE/ISCE Douro, 4560-408 Penafiel, Portugal; 5 Department of Sport Science, Polytechnic Institute of Bragança, 5300-252 Bragança, Portugal

**Keywords:** young people, exercise, physical fitness, motor proficiency, creativity

## Abstract

Physical Education plays a fundamental role in promoting healthy habits and lifestyles, as well as in the development of individual and cognitive skills. To date, several investigations have reported positive effects on indicators of physical fitness, motor proficiency, and creativity as a result of specific training programs during Physical Education classes. However, the effects of multivariate training programs on the improvement of the aforementioned skills remain unclear in the literature. Through this brief review, the benefit of applying multivariate training programs during Physical Education classes on indicators of physical fitness, motor proficiency and creativity was critically analyzed. A narrative approach was applied to summarize the availed research as following: (i) theoretical background; (ii) research gaps/issues; (iii) subject explanation about multivariate training programs in Physical Education; and (iv) practical application and further research. The evidence reported in this regard may be useful for the development of multivariate training programs that simultaneously enable the improvement of indicators of physical fitness, motor proficiency and creativity. However, there is still no consensus in the literature on the best strategies (i.e., type of program, duration, intensity) to enhance motor proficiency and creativity in the context of Physical Education classes using multivariate training programs.

## 1. Introduction

Physical education plays a fundamental role in the student’s integral development, enabling cognitive, psychomotor, and affective development, while stimulating healthy lifestyles, socialization, team spirit, and sports practice [1,2]. In fact, the benefits resulting from the regular practice of physical activity are diverse, with emphasis on improving cardiovascular and respiratory functions, reducing levels of anxiety and depression, increasing a sense of well-being, as well as developing cognitive and social skills [3,4]. In contrast, sedentary lifestyles have been associated with a decrease in functional abilities, an increase in morbidity and mortality, and an increase in the prevalence of chronic diseases in adulthood [5]. Thus, it is essential to promote healthy lifestyle habits and physical activity in childhood, which, extending from adolescence to adulthood, can play a key role preventing physical inactivity and have a beneficial influence on general health [6]. Nowadays, and due to the existence of an increasingly challenging and stimulating world, it would be expected that, from an early age, there would be a concern with the development of motor, social, and cognitive skills [7,8]. However, the reality is different, with exponential growth of sedentary lifestyle, interaction problems and reduced contact with nature, which puts the development of children’s motor skills at risk [9,10].

Particularly, with regard to Physical Education classes, one of the strategies that can increase their effectiveness, and which has been implemented in the school context, is the multivariate training program that, duly adapted, allows the inclusion of various content and the development of various individual skills, as well as improving the practice of physical exercise [10,11,12,13]. This methodology is applied in physical education classes with a view to aggregate strength and skill-based training, physical education-based interventions, sports based-training programs and physical activity-based lifestyle intervention [14,15,16]. However, a lack of evidence has been reported about the characteristics of the multivariate training programs due to the variability present in the application of the methods in different contexts [17]. Furthermore, in this sense, there are different methodological proposals, with different goals and duration that still do not reach a consensus on their application, therefore more research is needed on the subject. Multivariate training program have been conceptualized in some studies as a physical education-based strategy by applying an integrating evidence of teaching-learning methods for school-age children and youth.

This narrative review highlights the potential benefits of applying multivariate programs on physical fitness, motor proficiency and creativity in children and young people during Physical Education classes. Ultimately, a review is needed to summarize the findings and new evidence on the effects of applying multivariate programs in young populations. 

## 2. Materials and Methods

### 2.1. Literature Search Strategy

The Preferred Reporting Items for Systematic Reviews and Meta-Analyses (PRIS-MA) guidelines and the Population-Intervention-Comparators-Outcomes (PICOS) de-sign were followed to search the studies that were reviewed in current narrative review. To carry out this narrative review, the available literature was investigated by a structured and exploratory search into the Web of Science (Core Collection: Citation Indexes), PubMed, and SPORTDiscus electronic databases. Articles published in 2021 or earlier were considered. The search strategy comprised search words that combined one of two primary keywords (“multivariate training programs” and “physical education”), with a second keyword (“children”, “youth”, “physical fitness”) and a third keyword (“motor proficiency”, “creativity”), using the Boolean operator.

**Figure 1:**
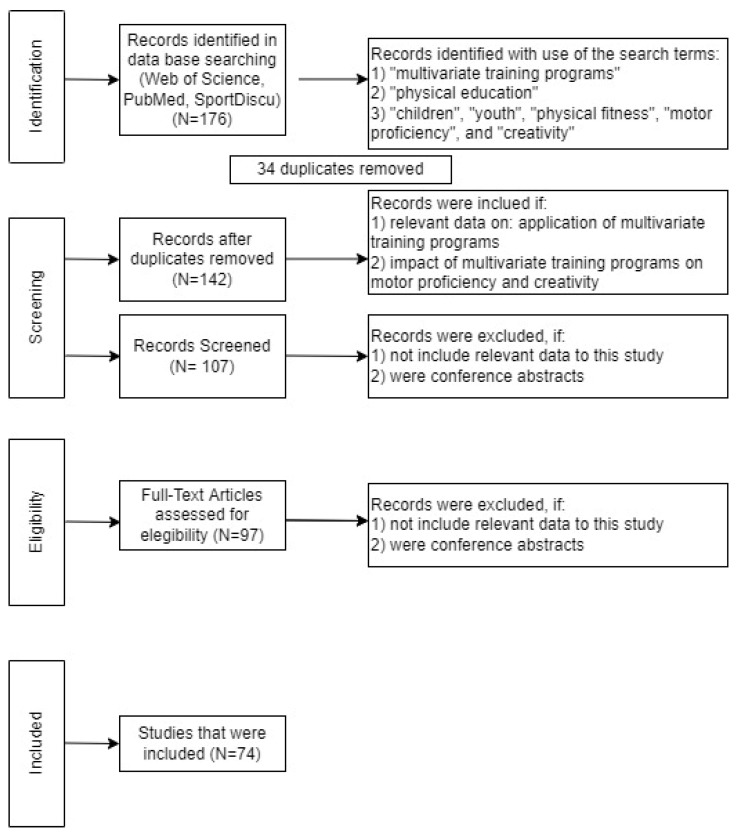
Prisma 2009 flow diagram.

The inclusion criteria for these articles were: (1) Relevant data on the application of multivariate training programs; (2) impact of multivariate training programs on motor proficiency and creativity. Studies were excluded if: (1) They did not include data relevant to this study; and (2) were conference abstracts. To assess the quality of the studies, a validated protocol was used [11,18]. The articles were screened based on the evaluation of the title and abstract. All articles that did not focus on the investigation were excluded. In total, 97 articles were considered relevant for this review. All articles have been read in detail and assessed for relevance and quality by two senior researchers with experience and relevant publications in the field. Discrepancies between the authors in the study selection were solved with support of a third reviewer. The authors did not prioritize authors or journals. All articles that did not meet the criteria were excluded. A total of 144 duplicate records were removed, and 100 articles were removed based on the title and abstract according to inclusion and exclusion criteria. After this procedure, 68 articles remained for analysis using PRISMA flow diagram (Figure 1).

### 2.2. Quality Assessment and Narrative Revision

Current narrative review was based on the methodological quality by the CONSORT stands for the Consolidated Standards of Reporting Trial [19]. A survey and narrative interpretation was subsequently carried out to scrutinize the theoretical considerations and future perspectives about multivariate training programs in physical education classes. The summary of previous research was compiled in: (a) Theoretical background; (b) research gaps and issues. *Physical Education classes* and *Multivariate Training Programs* were further analyzed to expose the explanation of subject matter, as well as the practical application and suggestions for further research. 

## 3. Summary of Previous Research

### 3.1. Theoretical Background

Regular participation in physical education classes has the potential to develop physically literate individuals and can stimulate skills and confidence to practice physical activity as a lifestyle option from an early age [12]. Furthermore, a considerable amount of research has indicated that physical activity in children can influence a range of physiological and psychological variables [13,14]. For this reason, there has been a growing interest in the knowledge derived from the application of multivariate training programs to children, emphasizing the significant benefits that have been reported previously [15,16]. In fact, according to previous research [17], as long as they are prescribed in sufficient quantity and with functional loads that allow them to exceed their usual muscle activity in accordance with the methodological recommendations for training in children and young people, these types of programs are beneficial for younger populations. However, previous investigations on the application of multivariate training programs in the context of physical education classes are inconclusive and show contradictory results [20,21,22,23,24,25,26]. In general, this type of program is composed of several stations that aim to enhance strength, balance, resistance, and coordination [26]. Strong et al. [20] denotes that the children and youth in school-age would participate 60 min per day or more in a moderate to vigorous intensity. Moreover, the authors have concluded that the physical activity must be developed in appropriate, enjoyable, and multiple activities [26]. Strength training sessions should be at least twice per week with non-consecutive sequence [20,21]. Hajihosseini [21] advocated that school-based interventions must be multi-component approach with a simultaneous targeting curricular, school environment and policy, as well as a community link for promoting physical activity and motor development. Kokkonen et al. [25] have mentioned that creative physical education-based approaches may increase students’ perceptions of task-supportive climate in physical activity, which predicts their later leisure-time physical activity motivation outside the school context and overall physical activity.

Recent studies indicate that children and adolescents can benefit from this type of program in a school context [2,9,15,27] and that its application can also enhance the development of motor proficiency [28] and of creativity simultaneously [22]. In this regard, several studies have concluded that physical exercise induces beneficial effects on cognitive processes [29,30,31] and that physical education classes are characterized as the ideal place to stimulate creativity in children [32]. Even so, the real impact of physical exercise on cognitive processes depends on several factors, including environment, typology, duration, and intensity [33,34,35]. A recent review [33] described the possible insights and strategies to be explored by teachers and educators in physical educations classes in the following vein: (i) flexible use of space and time; (ii) appropriate materials; (iii) working outside strategies for the classroom/school; (iv) ‘playful’ or ‘games-bases’ approaches with a degree of learner autonomy; (v) respectful relationships between teachers and learners; (vi) opportunities for peer collaboration; (vii) partnerships with outside agencies; (viii) awareness of learners’ needs; (ix) non-prescriptive planning. The review also described the impact of creative environments and the implementation of teaching-learning processes [34]. This becomes more important when considering that the exercise induced uncorrelated changes in cognition within mood or anxiety modifications which may suggest a separate effect for each component in the neural systems [36]. It is known that different types of exercise have different effects on cognition [36,37]; however, little is known about the most effective type of intervention and/or exercise to promote creativity in children and young people and this information is crucial and needs further clarification. In fact, this is valuable information for a more adequate planning of Physical Education classes and for the formulation of multivariate training programs that allow the simultaneous and effective development of motor proficiency, physical fitness, and creativity.

### 3.2. Research Gaps and Issues

Although one of the most important goals of physical education should be the development of motor skills [38,39] and physical fitness [40,41], it is also essential to improve cognitive processes and specifically creativity. Low competency in FMS was strongly associated with lower cardiorespiratory fitness and physical activity levels in childhood and adolescence [39]. Moreover, motor control and proficiency in childhood were more likely to become active adolescents and adult [38,40]. Motor skill development should be a key strategy in childhood interventions aiming to promote long-term physical activity [25,27,28]. In fact, evidence suggests that there is a causal relationship between physical fitness and brain vitality [42]. In particular, cardiorespiratory fitness and motor proficiency play an active role in cognitive development during childhood and youth [43], and for this reason, the increase of physical fitness can therefore be beneficial for the cognitive development of children and young people [44]. Furthermore, physical education programs also aimed for the health promotion through physical fitness, among which the improvement of the cardiometabolic indicators and muscle-skeletal health in students [40]. Nowadays, the environment created for children in a school context limits their creative potential, instead of stimulating their thinking, originality, curiosity, and daring [45]. In this sense, recent investigations have proposed principles that state that training programs for children and young people should follow the stimulation of the participants’ creativity [33,45,46]. Educational benefits of physical education and school sport have been extended to improve children’s concentration and arousal, which might indirectly benefit academic performance [47]. However, the interplay among the two learning contexts, physical education and sports, can be further explored. The literature is equally sparse in the use of qualitative methodology, something that can be a complement of greater depth and sensitivity in relation to quantitative data [3,11,24]. Quantitative and qualitative data make it possible to collect valid information for a pre- and post-program evaluation [21]. 

The impact of training programs applied in the context of Physical Education classes in improving motor skills and physical fitness is widely accepted in the literature; however, little is known about the potential of this type of programs in the development of creativity. Despite the apparent complexity of the concept, the results suggest that creativity is a disposition that can be improved by optimizing the environment and developing appropriate training programs [48,49], so the physical education class context seems to be the ideal environment. Few studies have examined the relationship between creativity and physical fitness [50]. Previous studies have shown that exercise, such as aerobic exercise, can enhance creativity [51,52,53,54,55,56] and this idea was supported by investigations that used Game-Based Programs in Physical Education classes [42,57]. Following another training approach, a previous study [52] carried out with elementary school students investigated the effects of applying a multivariate training program based on creative thinking, diversified practice, and physical and pedagogical literacy for 5 months and concluded that it was effective in enhancing children’s creativity, fundamental motor skills, agility, and speed [55,56]. Over decades, several teachers have applied different conceptions about teaching and learning in physical, however, multivariate training programs with a teacher-centered approach to a more student-centered was developed to promote problem-solving skills, critical and autonomous thinking [53,55]. Teaching Games for Understanding (TGfU) [53], teaching tactical creativity in sports [52,54], nonlinear pedagogy [48,53] and physical literacy [41,58] are topics that should be explored if one considers applying a multivariate training program.

## 4. Explanation of Subject Matter

### 4.1. Physical Education Classes

Physical education classes are a determining factor for motor development [50], given that in many cases this is the only place where children are exposed to the practice of vigorous physical activity. Therefore, classes must include stimuli that allow a large number of experiences, compatible with the child’s global development [1]. It is essential that Physical Education classes take place in favorable contexts that allow a high number of motor experiences that will enable a progressive development of the child’s motor behavior, which is why the teacher must ensure a progression in the complexity and diversification of the activities performed in class [27,47,59]. The development of physical fitness in a school context during Physical Education classes promotes health through changes in body composition, less susceptibility to diseases, and better physical condition [20]. In this regard, some studies have reported successful intervention programs in the context of physical education classes mainly through the application of strength training programs [15,60,61] In addition to improving motor skills and increasing muscle strength and endurance performance, frequent participation in a strength training program in young people triggers relevant health benefits [15,60,61], enhancing body composition and motor coordination [60]. Furthermore, this type of training [62] also improves mental health and muscle strength, recognized for having a positive association with school performance [63]. Considering the above benefits, there are several health-related recommendations that aim to increase the number of children and youth involved in training programs that incorporate muscle strengthening [64]. For all these reasons, muscle strength should be a priority in any sports development program [58].

Physical education and sport are expressed as positive contexts and experience in schools, leading to enjoyment, diversity, and engagement with an increase of the physical activity and participation [1]. Moreover, applying physical education programs during the elementary school years enhances the movement skill learning, fitness condition, cognitive such as creativity and critical thinking [34]. Galhahue and Donnelly [50] explores the relationship between content standard, performance standard, and performance benchmark. First, a behavior that expresses the progress up to the aim and the expected level of achievement expresses the performance’ benchmark and standard. Pesce et al. [34] mentioned that acute and submaximal exercise, performed by students during physical education classes, may facilitate memory storage. On this basis, the content standard is characterized by the baseline that the student should be a physically educated person [34,50]. Learning contexts vary profoundly according to the level of education, gender, and previous experiences [25,40]. Physical education is the gateway for the promotion of the appropriate levels of physical activity in childhood, with healthy habits and active lifestyles in adulthood being linked to childhood practice [25,27,41]. Indeed, Errisuriz et al. [27] emphasis the physical education-based interventions are a popular method to target children’s physical activity, body composition, and fitness. However, this only becomes effective empowering creative physical education, students’ perceptions, motivational climate, dynamical physical education lessons, and leisure-time physical activity [25,27].

Furthermore, creativity fostering classroom environment in elementary school plays a key role in creativity, critical thinking, and in future ability to make decisions and solve problems [46,52]. The interchange between physical education-interventions and sport-based training programs in creative behavior cannot be overlooked either [52]. Improving motor and cognitive development leads to children’s self-esteem, confidence, phonic knowledge, handwriting, and better engagement in sports [7]. Even more, psychomotricity and motor proficiency plays improve sports-related characteristics game-skilled improvements [52], as well as the competences in other areas of knowledge such as reading–writing and mathematical calculations [8]. The effects of gender in children’s cognitive and motor development were also reported in the literature [61]. Creating positive learning environments at physical education classes for female students using positive teaching strategies enables understanding the female students’ attitudes toward physical activity, sports performance, and participation/retention [59,61,65]. Evidence-based physical activity for school-age children and youth extends to several teaching methods such as strength and skill-based training [66], physical education-based interventions [21,23,52], sports based-training programs [2,52], and physical activity-based lifestyle intervention [17,20,21]. Thus, multivariate training programs have become increasingly important in the training of educators and teachers in the sense of aggregating all the approaches previously reported using multidisciplinary interventions [2,16].

### 4.2. Multivariate Training Programs

The literature has shown that multivariate training programs can be effective in promoting health and improving physical fitness indicators in children and young people [2,66]. In this regard, integrated neuromuscular training, which incorporates general activities (i.e., fundamental movements), specific activities (i.e., exercises to improve motor deficits), and strength and conditioning exercises (i.e., resistance, dynamic stability, plyometric and agility) has been recommended [67,68]. This type of approach allows children and young people to experience mastery of fundamental movement skills such as locomotion, stability, and manipulation skills [68]. Integrative training is defined by Myer et al. [68] as a multivariate training program or plan that incorporates general and specific strength and conditioning activities with congruent aims such as health- and skill-related components. Moreover, Fort-Vanmeerhaeghe et al. [68] expresses the need to apply an integrative neuromuscular training in order to improve injury resilience and to enhance sport and motor performance abilities in youth populations. This is a crucial point since the impact of a sedentary lifestyle during childhood and adolescence on lifelong pathological processes seems to extend to adulthood if unhealthy behaviors during this vulnerable period of life are not managed and prevented [68]. In addition, other investigations have also reported gains in muscle strength and improvements in movement mechanics [65,69]. This type of multivariate program has been recognized as an innovative approach [70] that can be implemented in a physical education classroom context [71]. A previous study reported improvements in fundamental motor skills and physical fitness after applying an integrated neuromuscular training program for 8 weeks, in the initial phase of the physical education class (i.e., 15 m) [70]. Similar conclusions were reported by a recent study [9] that investigated the effect of 10 weeks of integrated neuromuscular training in a school context, while another investigation [72] examined the effects of integrated neuromuscular training combined with yoga and varied stretching. 

Another multivariate training program was tested on young students during 20 physical education classes with the aim of developing creativity [22]. The applied training program consisted of exercises that: (i) Resorted to the use and modification of movement elements; (ii) developed creative thinking during movement activities through exploration; (iii) used movement to learn concepts from different subject areas of teaching; and (iv) developed critical thinking during movement activities. The results presented improvements in creative fluency as a result of participating in the training program during physical education classes. According to Nielson et al. [23], the acquisition of formation, new perspectives, and teaching methods for the physical education teachers can enable the implementation of the program. Indeed, Mura et al. [24] reports the schools as an ideal setting to implement physical activity programs in order to improve youths’ learning, intellectual abilities, and health habits. Concurrently, the multivariate training programs has been associated with improvement in cognitive skills and academic proficiency [42,43,47]. Several studies provided multiple intervention components also demonstrating improvements in children’s physical activity, fitness, and body composition, typically multiple components were implemented simultaneously [27]. Bailey et al. [47] suggest futures programs for physical education and school sport with the incorporation another evaluation research strategy as qualitative procedures. This would allow for an in-depth assessment of affective benefits [25,49]. Affective variables can be characterized as psychological, mental, and emotional well-being, being able to assess the mental health, positive self-regard, coping skills, conflict resolution skills, mastery motivation, a sense of autonomy, moral character, and confidence [47]. Aggregating psychomotor training programs with multivariate training programs can also explore motor proficiency and cognitive skills [26]. 

Although physical exercise has shown to be an effective tool in improving and developing students’ creativity [42,43,44], other multidisciplinary approaches have also shown to be effective [22,52]. Ultimately, there are different types of training programs that have shown to be effective for the individualized or simultaneous development of students’ physical and cognitive abilities [45,48]. However, focus of physical activity guidance and physical education classes on the exercise quantity may limit qualitative features for multivariate training programs such as the skill development, socialization, and exercise enjoyment [69]. The timing of brain development and the neuroplasticity associated with motor skill learning makes the pre-adolescent period a critical time to develop and strengthen fundamental movement skills in boys and girls [8,24,36]. Santos et al. [52] reports that he sports is an ideal environment for fostering creative behavior, arguing that a higher-order disposition can differentiate the everyday life of a child. Effectively, the creativity can be defined by a different dimensional definition such as strength, breadth, and depth [55]. Various types of training (e.g., aerobic, strength, circuit, flexibility and balance training), as well as sports- and physical activity-based intervention have reported a high capacity to enhance creativity and motor development [2,52,53,54,55]. For this reason, the type of training program most congruent to simultaneously develop creativity, motor proficiency and physical fitness in the context of Physical Education classes remains to be clarified.

## 5. Practical Application and Suggestions for Further Research

In general, previous studies have verified the benefits of applying different training programs in numerous variables (i.e., physical fitness, motor proficiency and creativity). Current data, however, show the lack of consensus on the best strategy to improve each of the variables, therefore, it needs to be clarified, as well as a multivariate training program that allows the simultaneous improvement of all variables investigated in this review (i.e., motor proficiency, physical fitness, and creativity) needs to be developed. Several authors have pointed out some suggestion for further research. Tomporowski et al. [42] recommended that a systematic examination should be carried out to analyze the type of physical activity in which children engage, and the task challenges that help understand the influence of cognitive development that occurs during physical activity. Ma et al. [49] denoted that meta-analysis about the scientific creativity theory is lacking. The authors underpin their assumptions arguing that the effectiveness of key components of training has been confirmed by creativity strategies, whereby not only will the training be more effective, but the process of creative thinking will be clearer. Otherwise, Rodriguez-Negro et al. [51] indicated that the short-term effects of different training lesson contents on motor and cognitive development should be studied using a long-term approach. Santos et al. [52] made several important points such as: (i) Coaches and educators may apply an enrichment training for children’s disposition and critical thinking; (ii) sport-based training is ideally suited for fostering creative behavior; (iii) training program possessed the fundamental motor and game-related skills. Tan et al. [53] presents various pathways for further research using TGflU and nonlinear pedagogy: (i) To study the relative effectiveness of teacher guidance on specific movement pattern; (ii) to analyze the affective (e.g., motivation) and physical (e.g., activity level) consequences of this approach to motor skills. Compiling these different teaching-learning methodologies may also represent new directions for understanding and creating new perspectives for the multivariate training program [2]. 

Following a new research trend, and to respond to gaps in the literature, future research can focus on multivariate training programs that allow the simultaneous development of physical fitness, motor proficiency, and creativity. Although there is agreement on the potential benefit of applying strength training programs in a school context, there is still no consensus in the literature on the best strategies (i.e., type of program, duration, intensity) to enhance motor proficiency and creativity in the context of physical education classes. It could be relevant to validate a multivariate training program that sought to answer open questions. Some of the findings may provide new insights for researchers and teachers to enhance the development of multivariate training programs in a physical education classroom context.

## 6. Conclusions

This study allows to conclude that multivariate training programs can be considered a valid strategy for physical education classes. Through this brief review, the benefit of applying multivariate training programs during physical education classes on indicators of physical fitness, motor proficiency and creativity were critically analyzed. The results found seem to indicate that this type of multivariate training programs could be used more effectively in a school context, and suggest that this type of training is a useful tool for the simultaneous development of physical fitness, motor proficiency, and creativity. However, there is still no consensus on specific recommendations for this type of programs and, therefore, further studies are still needed. In the same vein, future investigations should try to understand the differences during the application of multivariate training programs when applied in different contexts (i.e., school, leisure physical activity and sports environments).

## Data Availability

Not applicable.
